# Use of Emergency Department Extracorporeal Membrane Oxygenation for Treatment of Acute Necrotizing Myocarditis

**DOI:** 10.5811/cpcem.2018.11.40569

**Published:** 2019-01-04

**Authors:** Carly A. Loner, Peter W. Crane

**Affiliations:** University of Rochester Medical Center, Department of Emergency Medicine, Rochester, New York

## Abstract

We report a case of acute necrotizing eosinophilic myocarditis (ANEM) secondary to drug rash with eosinophilia and systemic symptoms (DRESS) related to administration of minocycline. Myocarditis is a rare complication of DRESS and can manifest as either a self-limited hypersensitivity myocarditis or as the frequently fatal ANEM. Due to the high morbidity and mortality caused by this disease, emergency physicians should be aware of the potential of ANEM in patients with history of DRESS and new-onset cardiac dysfunction. This case reviews the clinical presentation and management of ANEM and the potential role of extracorporeal membrane oxygenation use in the emergency department.

## INTRODUCTION

Drug rash with eosinophilia and systemic symptoms (DRESS) is a rare, drug-induced hypersensitivity syndrome most commonly associated with use of aromatic anticonvulsants and sulfonamides, but it can also occur with use of tetracyclines.^1^ Treatment of DRESS involves discontinuation of the offending drug and initiation of steroid therapy. Complications of DRESS include end organ dysfunction due to eosinophilic infiltration, including hepatic, renal, and cardiac dysfunction.^2^ The incidence of DRESS is one in 1,000–10,000 exposures with mortality estimated to be 10–20%, mainly caused by myocarditis and hepatic failure.^3^,^4^ The presentation of DRESS-associated myocarditis is delayed, presenting weeks to months after discontinuation of the causal drug.^5^ Most episodes of eosinophilic myocarditis are self-limited and will resolve with supportive therapy. However, some patients may develop a more severe form of myocarditis that involves eosinophilia and eventual necrosis of cardiomyocytes: acute necrotizing eosinophilic myocarditis (ANEM).^6^ This form of myocarditis can present with onset of rapidly deteriorating systolic dysfunction and hemodynamic instability.^5^

Emergency physicians should be aware of the potential of ANEM in patients previously diagnosed with DRESS. ANEM has a mortality rate of greater than 50% and a mean survival of three to four days.^5^ Precipitating factors include viral reactivation (human herpesvirus 6) and lack of detoxifying enzymes, permitting accumulation of toxic drug metabolites.^7^,^8^ Age may be a less important factor with ANEM occurring in patients ranging in age from 2–83 years old.^9^

Timely recognition of the potential diagnosis of ANEM is critical to starting appropriate therapy in these critically ill patients. The most common presenting symptoms are cardiogenic shock, hypotension, and chest pain.^10^ Few case reports in the emergency medicine literature depict the presentation of ANEM within the emergency department (ED), and even fewer detail the potential role of mechanical assist devices such as extracorporeal membrane oxygenation (ECMO) which are becoming increasingly prevalent within the ED.^3^,^5^,^11^ We present a case of ANEM secondary to DRESS after minocycline use, which required ECMO support due to cardiovascular collapse.

## CASE REPORT

A 21-year-old woman presented to the ED with complaint of chest pain and shortness of breath. Prior to arrival to the ED she had an episode of near syncope. Her previous medical history included development of diffuse erythematous rash following a course of minocycline prescribed for acne three months prior to ED presentation. The minocycline was discontinued, and she was treated with 30 milligrams (mg) daily oral prednisone with improvement of the rash. Initial vitals included blood pressure of 81/68 millimeters of mercury (mmHg), heart rate of 121 beats per minute (bpm), and respiratory rate of 18 breaths per minute. She was afebrile (36.7ºC oral temperature) and had pulse oximetry (SpO_2_) of 100% on room air. Physical exam was within normal limits. Electrocardiogram (ECG) showed right bundle branch block and normal ST-T segments, but no previous ECG was available.

While in the ED the patient had an episode of syncope during peripheral venous catheter placement, and intravenous (IV) fluids were administered due to concern of vasovagal event. She was also administered 5 mg IV dexamethasone due to possibility of adrenal suppression from steroid use. Her systolic pressure improved. However, the patient complained of worsening chest pain and then became unresponsive with pulseless electrical activity arrest (PEA). Cardiopulmonary resuscitation (CPR) and Advanced Cardiac Life Support were initiated. She received two doses of 1 mg IV epinephrine with return of spontaneous circulation (ROSC) in normal sinus rhythm of 70 bpm and blood pressure of 72/48 mmHg. Due to persistent hypotension, norepinephrine infusion was administered with improvement of blood pressure to 88/56 mmHg. She was intubated for airway protection.

Due to concern for massive pulmonary embolus, computed tomography chest angiography was performed but was unremarkable. Point-of-care echocardiogram demonstrated no right heart strain and grossly reduced heart function. Telemetry demonstrated QRS widening and increasing bradycardia to 41 bpm. The patient then developed a second PEA arrest with ROSC after CPR and one dose of 1 mg IV epinephrine. She remained hypotensive with blood pressure of 60/40 mmHg despite norepinephrine infusion. Initial troponin-T measured was 8.56 nanograms/milliliter (ng/mL) (reference range 0.0–0.02 ng/mL) and complete blood count with differential showed leukocytosis of 17.5 × 10^3^ cells/mL (reference range 4.0–10.0 × 10^3^ cells/mL) and eosinophilia of 1.6 × 10^3^ cells/mL r (reference range 0.0–0.4 × 10^3^ cells/mL). The cardiology service was consulted and a formal echocardiogram demonstrated a severely reduced ejection fraction of 15% (normal range 55–70%).

The dermatology service was also consulted at bedside in the ED; concern for ANEM in setting of DRESS given the rapidity of onset of her cardiac dysfunction was discussed. Also, the differential diagnoses included coronary vasculitis, viral myocarditis, infiltrative cardiomyopathy, and sepsis. (She was administered 4.5 g piperacillin/tazobactam and 20 milligrams/kilogram (mg/kg) vancomycin, and 500 mg azithromycin IV.)

While in the ED the patient developed further hypotension with blood pressure of 61/40 mmHg despite multiple vasopressors (IV vasopressin and norepinephrine infusions) and had severe acidemia and hypoxemia despite high ventilator support. Ventilator settings were tidal volume of 360mL, positive end-expiratory pressure of 20 centimeters of water (cmH_2_O), and fraction of inspired oxygen of 100%, and respiratory rate of 28 breaths per minute. Arterial blood gas (ABG) revealed respiratory acidosis with pH of 7.06, carbon dioxide partial pressure (pCO_2_) of 78 mmHg (reference range 33–43 mmHg), low arterial oxygen (PaO_2_) of 61 mmHg (reference range 80–100 mmHg), and normal bicarbonate. Basal metabolic panel was within normal limits. Due to continued decline, she received cannulation for veno-arterial extracorporeal membrane oxygenation (VA-ECMO) within the ED. ECMO cannulation occurred within three hours of initial cardiac arrest. Once placed on ECMO she had significant improvement in her acidosis and hypoxemia on repeat ABG (pH 7.39, pCO_2_ 41 mmHg, PaO_2_ 135 mmHg) and was rapidly weaned from her vasopressors.

**CPC-EM Capsule**

What do we already know about this clinical entity?

*Acute necrotizing eosinophilic myocarditis (ANEM) is a rare complication of drug rash with eosinophilia and systemic symptoms. Presenting symptoms are cardiogenic shock, hypotension, and chest pain*.

What makes this presentation of disease reportable?

*Few case reports depict the presentation of ANEM in the emergency department (ED). We review the potential role of mechanical assist devices such as extracorporeal membrane oxygenation (ECMO) to manage ANEM*.

What is the major learning point?

*ECMO is a potential therapy for cardiogenic shock resulting from ANEM. Early use of ECMO can preserve cardiac function and improve survival in these critically ill patients*.

How might this improve emergency medicine practice?

*This case highlights the presentation and management of ANEM, an emergent cause of cardiovascular compromise, and the use of ECMO in the ED*.

The patient was admitted to the cardiac intensive care unit (ICU) for further management. Due to concern of DRESS-induced myocarditis, dexamethasone with a dose of 1 mg/kg/day was administered intravenously. On hospital day (HD) eight, cardiac biopsy demonstrated diffuse active myocarditis with coagulative myocyte necrosis and mixed infiltrate including eosinophils consistent with DRESS myocarditis. She was decannulated from ECMO on HD 16. She was eventually discharged after 20 days in the ICU and 62 total days in the hospital with a life vest and continued cardiac follow-up. An echocardiogram performed three months later demonstrated improved ejection fraction of 34%.

## DISCUSSION

This patient had cardiovascular collapse soon after presentation to the ED due to ANEM from minocycline-induced DRESS. This was caused by eosinophilic infiltration of the myocardium and eosinophilic degranulation, which caused necrosis and apoptosis of cardiomyocytes.^6^ Histology confirmed the diagnosis of ANEM with diffuse eosinophilic myocarditis with lymphocytic infiltrate and liquefactive necrosis.^5^ Patients may present with chest pain and hemodynamic instability. Additional findings include ST-T segment elevation on ECG, elevated troponin, normal coronary arteries on angiogram, and rapidly deteriorating systolic function.^5^,^11^ Cardiac echocardiogram will typically show increased wall thickness, severe biventricular failure and pericardial effusion.^5^ DRESS-induced myocarditis, especially ANEM, is an emergent diagnosis that can cause refractory cardiovascular shock and may require mechanical cardiac support if less-invasive measures are unsuccessful.

Treatments commonly used in the treatment of DRESS-induced myocarditis include high dose systemic steroids (from 1 mg/kg to 1 gram IV daily), IV immunoglobulin, and mycophenolate mofetil.^5^ These treatments will limit eosinophilic infiltration into myocyte tissue and prevent degranulation.^6^ Plasmapheresis and immune suppressive agents such as mycophenolate mofetil, rituximab, and azathioprine can be used in conjunction with systemic steroids.^6^ These methods of treatment may be more appropriate in the setting of non-necrotizing hypersensitivity myocarditis, which often presents as slow-onset heart failure with improved hemodynamic stability in comparison to ANEM.^6^ In these cases, fluid restriction, angiotensin-converting enzyme inhibitors, beta blockers and diuretics can be used to prevent further decompensation.^6^

In the setting of cardiovascular shock, ECMO is an alternative therapy that can bypass the lungs and heart to support gas exchange and circulatory perfusion.^12^ There are two classifications of ECMO with different indications for initiation. VA-ECMO is used in cases of treatment-refractory cardiac failure or combined heart and lung failure to maintain systemic perfusion.^12^ In VA-ECMO, the venously drained blood is oxygenated extracorporeally, bypasses pulmonary circulation, and is returned to the aorta. Indications for VA-ECMO include ventricular dysrhythmias, pulmonary embolism, right and left ventricular failure, sepsis, and cardiac arrest.^12^,^13^ In contrast, venovenous ECMO (VV-ECMO) is indicated in severe hypoxemic respiratory distress. Venously drained blood is oxygenated and decarboxylated extracorporeally and returned to the right atrium. Indications for VV-ECMO include acute respiratory distress syndrome and hypercapnic failure.^12^,^13^

The time of initiation to ECMO is dependent on the rapidity of onset of systolic dysfunction, and earlier initiation may be associated with improved outcomes. The CHEER trial (mechanical CPR, hypothermia, ECMO, and early reperfusion) promotes the early use of ECMO in the setting of refractory cardiac arrest and cardiovascular shock. In this study, selected patients presenting with out-of-hospital or in-hospital cardiac arrest were started on ECMO with CPR if they had refractory cardiac arrest for greater than 30 minutes.^14^ ROSC was achieved in 92% (25/26) of patients, and the difference in median time of collapse to initiation of ECMO between survivors and non-survivors was 40 minutes compared to 78 minutes.^14^ Another study showed that ECMO use in pediatric patients with hemodynamic compromise from dysrhythmias of acute fulminant myocarditis had shorter times to recovering sinus rhythm (median time: 1.7 days vs. 7.35 days).^15^ ECMO has an important role in the treatment of refractory cardiovascular shock in patients with fulminant myocarditis.^14^,^16^,^17^ Early use of ECMO can preserve cardiac function and improve survival and morbidity.^14^,^15^,^17^ A multicenter, retrospective chart review would be beneficial to better quantify the benefits of ECMO use in DRESS-induced myocarditis in comparison to other therapies.

## CONCLUSION

Emergency physicians should be aware of the potential of ANEM in patients with new-onset cardiac dysfunction and history concerning for DRESS. ECMO is becoming increasingly prevalent, with studies showing reduced morbidity and mortality with earlier ECMO initiation. In some Level I trauma settings cannulation for ECMO may occur within the resuscitation bay of the ED. It is important for ED providers to be aware of the generalized types of ECMO and their indications. Timely recognition of the potential diagnosis of ANEM is critical to starting appropriate therapy. Early use of ECMO can preserve cardiac function and improve survival in these critically ill patients.
